# The Missing Process Hygiene Criteria in the Game Meat Chain: Challenges in Wild Boar Meat Production

**DOI:** 10.1155/vmi/5942428

**Published:** 2026-05-14

**Authors:** Laura Andriani, Mauro Conter, Martina Rega, Silvia Bonardi, Antonio Poeta, Giovanni Maria Pisani, Cristina Bacci

**Affiliations:** ^1^ Università Degli Studi di Parma Dipartimento di Scienze Medico-Veterinarie, Parma, Emilia-Romagna, Italy; ^2^ Azienda Unità Sanitaria Locale (AUSL), via Amendola 2, Reggio Emilia, 42122, Italy

**Keywords:** antimicrobial resistance, food hygiene criteria, foodborne pathogens, game meat supply chain, wild boar meat

## Abstract

Wild boars are the most widespread large wild mammals globally, capable of reaching high population densities due to their ecological adaptability and human‐related factors. This leads to significant environmental, economic, and public health issues, making effective management essential. Hunting remains the most efficient control strategy and offers the potential to develop a game meat supply chain. However, wild boars are known carriers of foodborne pathogens and antimicrobial‐resistant bacteria, and various hunting‐related factors can influence carcass hygiene. This study aimed to assess microbial hygiene indicators—aerobic colony count (ACC), *Enterobacteriaceae* count (EntC), *Salmonella*, and *Escherichia coli*—in wild boars hunted in the Emilia‐Romagna, northern Italy, during the 2022/2023 and 2023/2024 hunting seasons. A total of 115 sponge samples from carcasses and 78 diaphragm muscle samples were collected from eight game‐handling establishments (GHEs), with biometric and process‐related data also recorded. Results showed average ACC and EntC levels of 4.22 and 2.27 log CFU/cm^2^, respectively. *Salmonella* was found in 3.48% of samples, with limited resistance detected (only one isolate resistant to sulfamethoxazole). *E. coli* was detected in 97.43% of the samples, with no resistance observed to third‐generation cephalosporins or carbapenems. Of these *E. coli* isolates, 39.5% harboured at least one virulence gene, and the majority were classified as atypical enteroaggregative *E. coli* (aEAEC). The study highlighted that time intervals between killing, evisceration, and skinning had a significant impact on hygiene, recommending completion of all steps ideally within 4 h. Although specific hygiene criteria for wild game are lacking, the observed microbial levels were within acceptable limits set for livestock by Regulation (EC) No 2073/2005. The findings underscore the need for standardized processing practices to ensure safety in wild boar meat production.

## 1. Introduction

Pigs are one of the few domesticated species whose wild counterparts, wild boars (*Sus scrofa*), continue to thrive in natural environments. Wild boars are one of the most widespread large mammals globally. The ecological plasticity and high reproductive potential enable them to reach high population densities within a very short period of time, resulting in numerous economic, environmental, and social problems [1].

Nevertheless, in Europe, increasing numbers of wild boar sightings were reported also in urban and suburban areas, for instance, in Berlin, Barcelona, Rome, Vilnius, Budapest, Genoa, and Warsaw [2] particularly due to food scarcity or extreme temperatures in their natural environments [3].

In Italy, wild boars have rapidly expanded their range over the past 50 years. Currently, the Italian Institute for Environmental Protection and Research (ISPRA) estimates the wild boar population in Italy to be approximately 1.5 million individuals [4].

Wild boars pose significant conflicts with human activities and well‐being, including the risk of disease transmission to humans, livestock, and other domestic animals [5].

Management strategies for wild boar populations are diverse [6] and the most significant factor limiting wild boar distribution is hunting pressure, highlighting the necessity for effective hunting management to control their populations [7].

As a result, the availability of meat from culled wild ungulates is increasing rapidly. In Italy, recent estimates indicate that the annual consumption of meat from harvested ungulates per capita is very low, ranging from 0.1 to 0.3 kg. However, data change when considering the number of hunters, who represent the most engaged consumer category reaching 1.0–4.0 kg annual per capita [8].

Although the organoleptic and microbiological quality of wild boar meat can be excellent, it varies significantly based on the conditions under which the animals are hunted, handled, and processed. The European regulations, including Regulation (EC) No 178/2002 [9], establish proper hygiene practices and delineate the responsibilities of hunters concerning meat safety. These regulations also mandate official veterinary inspections at designated game‐handling establishments (GHE) and ensure the traceability of game meat [9–12]. However, considering the natural behaviour of wild boars and the numerous variables associated with the hunting process, the risk of foodborne diseases linked to the consumption of wild ungulate meat cannot be entirely eliminated. Furthermore, Regulation (EC) No 2073/2005 [13], which establishes microbiological criteria for certain microorganisms in foodstuffs, does not specify criteria for hunted wild game carcasses and meat [14].

In Italy, the State‐Regions Conference has established hygiene guidelines for wild boar meat, recognizing the significant availability of wild game and consumer demand. These guidelines aim to facilitate the development of supply chains for wild game meat, ensuring that quality and safety standards are met [15]. However, a clear and applicable national or regional legal framework has yet to be established, which hampers the development of a robust game meat supply chain. Indeed, hunters who utilize game meat for personal consumption or sell it directly to end consumers or local retailers [16] are exempt from these regulations, but still adhering to traceability standards. Additionally, the traders who purchase game from hunters must keep records documenting the product’s origin, in compliance with Regulation (EC) No 178/2002 [9, 17].

The legislation at all levels fails to designate an institutional authority responsible for ensuring the safety of game products intended for personal consumption or direct sale. This absence of comprehensive oversight throughout the production chain by a competent authority raises significant sanitary concerns [18].

Notably, wild boars act as reservoirs for foodborne zoonotic pathogens, as for *Salmonella* spp. and pathogenic strains of *Escherichia coli* [19].

*Salmonella* spp. is one of the most frequently identified gastrointestinal pathogens in humans across the European Union (EU) [20]. Transmission primarily occurs through the oral–faecal route, involving the consumption of contaminated food or water, direct contact with infected animals, or exposure to contaminated environments [21]. Game meat was identified as a minor source, but the increase in the availability of wild boar meat in the market underscores the need for systematic and ongoing monitoring of *Salmonella* presence, employing a risk‐based approach for its prevention and control [20].

Wild boars can serve also as significant reservoirs for *Escherichia coli*, including pathogenic strains. Like other members of the *Enterobacteriaceae* family, these bacteria primarily inhabit the digestive tracts of animals [22, 23], and it is considered a Process Hygiene Indicator by European legislation.

Pathogenic *E. coli* is currently divided into distinct pathotypes that are associated with gastrointestinal infections, classified based on their virulence genes and mechanisms of pathogenicity. They include enteropathogenic *E. coli* (EPEC), Shiga toxin‐producing *E. coli* (STEC) or verocytotoxin‐producing *E. coli* (VTEC), enteroinvasive *E. coli* (EIEC), enteroaggregative *E. coli* (EAEC), enterotoxigenic *E. coli* (ETEC), and atypical *E. coli* [20].

In Europe, various studies indicate that wild boars serve as reservoirs for STEC and EPEC. The prevalence and pathotypes of these pathogens vary based on local conditions, hunting practices, and the type of sample collected [24].

Another potential risk associated with consuming contaminated game meat is the emergence of antimicrobial‐resistant strains. Antimicrobial resistance (AMR) has existed for millions of years as an inevitable evolutionary consequence of microbial competition in natural environments.

Globally, AMR poses a significant challenge in both human and veterinary medicine, obstructing the effective treatment of bacterial infections, and it is estimated to cause 25,000 deaths annually in Europe [19].

Antimicrobial‐resistant bacteria can enter the food chain through antibiotic use in aquaculture, livestock, and crop farming. Environmental factors contribute as well, allowing resistant strains to spread throughout food production stages [25]. Notably, AMR is no longer confined to livestock and human environments; it has become a wider environmental concern with substantial public health implications [26]. Globally, AMR is increasing, with wildlife serving as a reservoir for clinically significant resistance determinants. Some studies have shown that wildlife populations living near human or agricultural areas exhibit higher levels of AMR compared to those in more remote or natural settings. Wild boars constitute a reservoir for antimicrobial‐resistant bacteria and may function as sentinel species [26].

This study examines the microbiological contamination of wild boar carcasses hunted in the Emilia‐Romagna region (Italy), with a focus on the process hygiene criteria, i.e., aerobic colony count (ACC), *Enterobacteriaceae* count (EntC), and the detection of *Salmonella* spp. Additionally, the study evaluates *E. coli* as a hygiene indicator in the diaphragm muscles of the same wild boars, analyzing its prevalence, AMR, and pathotypes. The influence of biometric data and specific hunting process variables on contamination was also investigated, providing meaningful insights into the potential health risk associated with the consumption of wild boar meat.

## 2. Materials and Methods

### 2.1. Samples Collection

**FIGURE 1:**
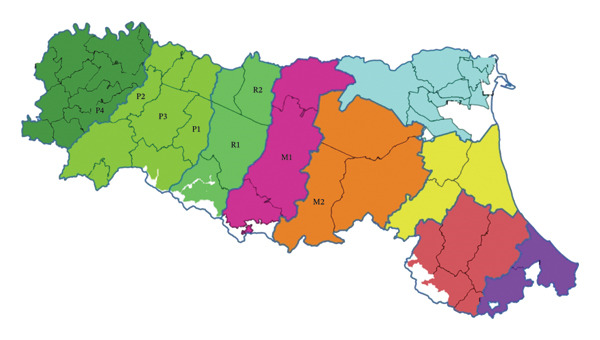
Geographical distribution of ATCs and relative GHEs in Emilia‐Romagna region (Regione Emilia Romagna‐agricoltura, caccia e pesca https://agricoltura.regione.emilia-romagna.it/fauna-e-caccia/caccia/atc).

During the 2022/2023 and 2023/2024 hunting seasons, a total of 115 sponge samples from wild boar carcasses and 78 diaphragmatic muscle samples from the same animals were collected across eight GHEs, designated as R1, R2, M1, M2, P1, P2, P3, and P4, in Emilia‐Romagna, Italy. The wild boars sampled were harvested by local hunters in designated hunting areas (ATCs) within the provinces of Parma, Reggio‐Emilia, Modena, Bologna, and Piacenza, and transported to the nearest GHE (see Figure 1) [27].

According to the ISO 17604:2005 method for swine, the sponge procedure was conducted at four specific points (loin, jowl, medial surface of the leg, and belly) on wild boars’ carcasses, after evisceration and skinning, covering a total area of 400 cm^2^. Diaphragmatic muscle samples were also collected, while the remaining parts of the carcasses were left at the disposal of hunters for subsequent consumption. However, it was not possible to collect diaphragmatic muscle samples from all wild boar carcasses. The wild boars biometric data, along with information on variables associated with the hunting process, were recorded including: (i) GHE of destination; (ii) sex; (iii) age (young or adult); (iv) hunting methods (drive hunting or “girata”); (v) shot placement accuracy; (vi) presence of pathological alterations; (vii) time period between killing and evisceration; (viii) time period between evisceration and skinning; (ix) temperature of the refrigeration rooms at the GHEs.

Shot accuracy was deemed correct if it did not occur in the abdominal area, which could result in intestinal rupture.

The samples were transported as promptly as possible, under refrigerated conditions (4 ± 1°C), to the laboratory of Inspection of Animal‐Origin Food, University of Parma (Italy). If not processed immediately, they were stored under refrigeration (4 ± 1°C) and processed within 24 h.

### 2.2. ACC and EntC

ACC and EntC were initially performed according to ISO 6887‐1:2017 [28], with each sponge sample diluted 1:10 in 90 mL of buffered peptone water (BPW‐Oxoid Ltd., Basingstoke, UK), followed by serial decimal dilutions in BPW up to 10^−7^ for ACC and 10^−6^ for EntC. For ACC, 1 mL of each dilution was plated, in duplicate, on Plate Count Agar (PCA‐Oxoid) plates and incubated at 30°C ± 1°C for 72 h [29]. For EntC, 1 mL of each dilution was plated, in duplicate, on Violet Red Bile Glucose Agar (VRBGA‐Oxoid) plates, and incubated at 37°C ± 1°C for 24 h [30]. Following the incubation period, colonies were counted. Regarding EntC, five colonies were selected from each counted plate, and biochemical confirmations were performed using oxidase reaction and glucose fermentation tests. In accordance with the ISO 7218:2013 [31] guidelines, count formula was applied for both ACC and EntC, with results expressed as colony‐forming units per square centimetre (CFU/cm^2^). The results were then adjusted to the measured area of 400 cm^2^.

### 2.3. Detection and Serotyping of *Salmonella* spp.

The same sponge samples, previously diluted in BPW, were incubated at 37 ± 1°C for 18 ± 1 h as a pre‐enrichment step for the detection of *Salmonella* spp. [32]. The samples were then inoculated into selective enrichment broths: MullerKauffman Tetrationate Novobiocin Broth (Biolife, Milan, Italy) and Rappaport‐Vassiliadis Broth (Oxoid), and incubated at 37 ± 1°C and 41 ± 1°C, respectively, for 24 h. Ten microlitre aliquots of the enrichment broths were plated onto selective and differential agar media, specifically Xylose Lysine Deoxycholate (XLD) agar (Oxoid) and Chromogenic *Salmonella* Agar Esterase (Neogen, Lansing, Michigan, USA), and incubated at 37 ± 1°C for 24 h. The colonies, exhibiting typical *Salmonella* spp. morphology, were subjected to biochemical and serological confirmation tests and subsequently identified using the API 20E microsubstrate system (bioMérieux, Lyon, France). *Salmonella* isolates were serotyped in accordance with the ISO/TR 6579‐3:2014 [33] method by the Istituto Zooprofilattico Sperimentale della Lombardia ed Emilia‐Romagna.

### 2.4. *Salmonella* AMR Evaluation

AMR of the *Salmonella* strains was evaluated by determining the minimum inhibitory concentration (MIC) using the Sensititre EU Surveillance *Salmonella/E. coli* Plate (Thermo Fisher Scientific, Waltham, Massachusetts, USA), following the manufacturer’s instructions. Each plate was customized with the following antibiotics (μg/mL): (i) β‐lactams: ampicillin (AMP 1–32), cefotaxime (CTX 0.25–4), ceftazidime (CAZ 0.25–8), meropenem (MEM 0.03–16); (ii) macrolids: azithromycin (AZI 2–64); (iii) aminoglycosides: amikacin (AMI 4–128), gentamicin (GEN 0.5–16); (iv) quinolones/fluoroquinolones: nalidixic acid (NAL 4–64), ciprofloxacin (CIP 0.015–8); (v) polymixin: colistin (COL 1–16); (vi) glycylcycline: tigecycline (TGC 0.25–8); (vii) sulphonamides: trimethoprim (TMP 0.25–16), sulfamethoxazole (SMX 8–512); (viii) tetracyclines (TET 2–32); (ix) chloramphenicol (CHL 8–64).

European Committee on Antimicrobial Susceptibility Testing (EUCAST) [34] and Clinical & Laboratory Standard Institute (CLSI) [35] guidelines were applied to classify MIC values as resistant, intermediate, or susceptible.

### 2.5. *E. coli* Isolation and AMR Evaluation

*E. coli* isolation was performed in wild boar diaphragmatic muscle (25 g). Each sample was diluted 1:10 with 225 mL of BPW (Oxoid) and incubated at 37°C ± 1°C for 20 h. Following incubation, 20 μL of the enriched sample was plated onto Tryptone Bile X‐glucuronide agar (TBX) (Oxoid) and incubated at 41°C ± 1°C for 24 h. A single isolated colony per sample exhibiting typical *E. coli* morphology was selected. The colonies were then seeded onto Tryptic Soy Agar (TSA) (Oxoid) and incubated at 37°C ± 1°C for 24 h for oxidase and indole testing. Biochemical identification was subsequently performed using the API 20E microsubstrate system (bioMérieux).

*E. coli* isolates were tested for AMR against cefotaxime (CTX 5 μg), ceftazidime (CAZ 10 μg), and meropenem (MEM 10 μg) using the Kirby‐Bauer disk diffusion method, following EUCAST [34] guidelines.

### 2.6. Detection of *E. coli* Virulence Genes

Two multiplex end‐point PCR assays were used for the detection of *E. coli* typical pathotype genes. DNA from *E. coli* isolates was extracted using the heat‐based lysis method. Specifically, three colonies grown on Tryptic Soy Agar (TSA‐Oxoid) were suspended in 1 mL of sterile distilled water, subjected to heating at 95°C for 10 min, and the cellular debris was removed by centrifugation at 15,000 RCF (ALC microCentrifugette 4214) for 5 min. The DNA in the supernatant was quantified using a NanoDrop spectrophotometer (Thermo Fisher Scientific), and subsequently used as a template for PCR amplification. The first multiplex PCR assay was used to detect the *stx1* and *stx2* genes, characteristic of STEC, and the *eae* gene, which is typically associated to EPEC and STEC. The primer sequences, PCR mixture, and PCR cycling conditions were applied in accordance with Annex C of the ISO/TS 13136:2012 [36] (Table 1). The PCR mixture, prepared to a final volume of 50 μL per sample, consisted of 1x Green GoTaq Flexi Buffer, 2 U of GoTaq G2 Flexi DNA Polymerase, 1.2 mM of MgCl_2_ (Promega, Wisconsin, USA), 0.2 mM of each dNTPs (Promega), primers at concentration of 0.25 μM, 10 μL of DNA sample template, and nuclease free water to reach the final volume.

**TABLE 1:** Conditions for the first multiplex PCR assay used to detect *E. coli* typical pathotype genes, as described in the ISO/TS 13136:2012 [36].

Pathotype	Target gene	Primer sequences	Size (bp)	PCR cycling conditions
STEC	*stx1*	F 59‐ATAAATCGCCATTCGTTGACTAC‐39 R 59‐AGAACGCCCACTGAGATCATC‐39	180	35 PCR cycles, each consisting of 1 min of denaturation at 95°C; 2 min of annealing at 65°C for the first 10 cycles (decreasing by 1°C per cycle to 60°C from cycles 11–15); and 1.5 min of elongation at 72°C (extended to 2.5 min from cycles 25–35).
	*stx2*	F 59‐GGCACTGTCTGAAACTGCTCC‐39 R 59‐TCGCCAGTTATCTGACATTCTG‐39	255	
EPEC, STEC	*eae*	F 59‐GACCCGGCACAAGCATAAGC‐39 R 59‐CCACCTGCAGCAACAAGAGG‐39	384	

The second multiplex PCR assay targeted several genes: *escV*, characteristic of LEE‐positive strains; *bfpB*, specific to EPEC; *elt*, *estIa*, and *estIb*, associated with ETEC; *invE*, a marker for EIEC pathotype; *astA*, *aggE*, and *pic* of EAEC; *uidA* gene, a housekeeping gene in *E. coli* encoding the beta‐glucuronidase enzyme, which was used as an internal PCR control. The primer sequences, PCR mixture, and PCR cycling conditions used were adapted from Müller et al. [37] (Table 2). The PCR mixture was prepared to a final volume of 25 μL per sample, including: 1x Green GoTaq Flexi Buffer; 2 U of GoTaq G2 Flexi DNA Polymerase; 2.1 mM of MgCl_2_ (Promega); 0.3 mM of each dNTP (Promega); primers for *escV*, *estIa*, and *astA* at 0.4 μM; primers for *estIb*, *invE*, *aggR*, *pic*, and *uid* at 0.2 μM; primers for *elt* and *bfpB* at 0.1 μM; and 4.75 μL of DNA template.

**TABLE 2:** Conditions for the second multiplex PCR assay used for the detection of *E. coli* typical pathotype genes [37].

Pathotype	Target gene	Primer sequences	Size (bp)	PCR cycling conditions
STEC, EPEC	*escV*	F 59‐ATTCTGGCTCTCTTCTTCTTTATGGCTG‐39 R 59‐CGTCCCCTTTTACAAACTTCATCGC‐39	544	5 min of initial denaturation, 30 cycles, each consisting of 30 s of denaturation at 95°C; 30 s of annealing at 63°C; and 1.5 min of elongation at 72°C; 5 min of final elongation at 72°C.
EPEC	*bfpB*	F 59‐GACACCTCATTGCTGAAGTCG‐39 R 59‐CCAGAACACCTCCGTTATGC‐39	910	
ETEC	*elt*	F 59‐GAACAGGAGGTTTCTGCGTTAGGTG‐39 R 59‐CTTTCAATGGCTTTTTTTTGGGAGTC‐39	655	
	*estIa*	F 59‐CCTCTTTTAGYCAGACARCTGAATCASTTG‐39 R 59‐CAGGCAGGATTACAACAAAGTTCACAG ‐39	157	
	*estIb*	F 59‐TGTCTTTTTCACCTTTCGCTC‐39 R 59‐CGGTACAAGCAGGATTACAACAC‐39	171	
EIEC	*invE*	F 59‐CGATAGATGGCGAGAAATTATATCCCG‐39 R 59‐CGATCAAGAATCCCTAACAGAAGAATCAC‐39	766	
EAEC	*astA*	F 59‐TGCCATCAACACAGTATATCCG‐39 R 59‐ACGGCTTTGTAGTCCTTCCAT‐39	102	
	*aggR*	F 59‐ACGCAGAGTTGCCTGATAAAG ‐39 R 59‐AATACAGAATCGTCAGCATCAGC‐39	400	
	*pic*	F 59‐AGCCGTTTCCGCAGAAGCC‐39 R 59‐AAATGTCAGTGAACCGACGATTGG‐39	1111	
Internal control	*uid*	F 59‐ATGCCAGTCCAGCGTTTTTGC‐39 R 59‐AAAGTGTGGGTCAATAATCAGGAAGTG‐39	1487	

The PCR amplicons were evaluated by electrophoresis on a 2% agarose gel stained with SYBR Safe DNA gel stain (Invitrogen, Carlsbad, CA, USA) and visualized using a UV transilluminator.

A 100 base pairs DNA ladder (Promega) served as a molecular weight marker. *E. coli* ATCC 43894 served as positive control in the first PCR assay, while both negative and no‐template controls were included in all PCR assays.

### 2.7. Statistical Analysis

The ACC and EntC values were converted to Log CFU/cm^2^ for statistical analysis. Mean, standard deviation, median, quartiles (Q1, Q2, Q3, Q4), interquartile range (IQR), maximum, and minimum values were calculated for the ACC and EntC distributions. Data points outside the range defined by minimum > Q1 − 1.5 IQR and maximum < Q3 + 1.5 IQR were considered outliers. Normality of the ACC and EntC distributions was assessed using the *z*‐score, calculated by dividing the skewness values by their standard errors [38]. ACC and EntC values were analyzed separately, and statistically significant differences between count values and each recorded variable were assessed using one‐way ANOVA. Tukey–Kramer’s test was subsequently applied to identify significant differences between groups.

The prevalence of *Salmonella* spp. was evaluated. Statistically significant differences between samples with *Salmonella* detection and each recorded variable were assessed using the Chi‐squared test or Fisher’s exact test, depending on the number of observations in each category. A Mann–Whitney *U* test was then used to compare EntC values between samples with and without *Salmonella* detection.

The prevalence of *E. coli* was determined, along with the prevalence of virulence genes in the *E. coli* isolates, which were used to evaluate the probable associated pathotype. Statistical analyses were performed using Microsoft Excel.

## 3. Results

### 3.1. Wild Boar Samples and Hunting Variables

The 115 sponge samples collected from wild boar carcasses were analyzed for ACC, EntC, and *Salmonella* spp. The 78 diaphragmatic muscle samples were analyzed for the *E. coli* detection. No pathological alterations were observed in any of the wild boar carcasses. The temperature in the refrigeration rooms of the GHEs was always maintained below 7°C. Other variables considered in the study, including biometric data, hunting methods, shot placement accuracy, and the time intervals between killing, evisceration, and skinning, could not be fully recorded for every sample as detailed in Table 3.

**TABLE 3:** Number of wild boars for each biometric data and associated hunting process variables.

GHE	Sex	Age	Hunting method	Shot accuracy	Killing‐ evisceration time	Evisceration‐skinning time	Killing skinning time
R1:15 R2:7 M1:7 M2:3 P1:31 P2:29 P3:16 P4:7	Female: 43 Male: 49 No data: 23	Young: 45 Adult: 43 No data: 27	Driven‐hunting:54 Girata: 29 No data: 32	Yes: 93 No: 11 No data: 11	< 3 h: 79 ≥ 3 h: 29 No data: 7	< 10 h: 108 ≥ 10 h: 7	< 3.5 h: 101 ≥ 3.5 h: 14
115							

### 3.2. ACC and EntC Values in Relation to the Recorded Variables

**FIGURE 2:**
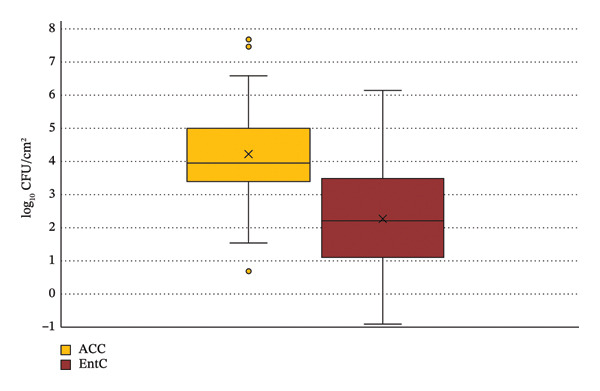
Distribution of ACC and EntC values, illustrating medians, means, interquartile ranges, extreme values, and outliers.

The ACC values ranged from a minimum of 0.7 Log CFU/cm^2^ to a maximum of 7.76 Log CFU/cm^2^, with a median of 3.97 Log CFU/cm^2^ and a mean of 4.22 Log CFU/cm^2^ (standard deviation of 1.27). The EntC values ranged from −0.9 Log CFU/cm^2^ to 6.63 Log CFU/cm^2^, with a median of 2.21 Log CFU/cm^2^ and a mean of 2.27 Log CFU/cm^2^ (standard deviation of 1.46). ACC values outside the range of 1.15 Log CFU/cm^2^ to 7.17 Log CFU/cm^2^ were identified as outliers. No outliers were observed in the EntC values (Figure 2). ACC and EntC values followed a normal distribution, as evidenced by *z*‐score < 3.29 for both distributions, corresponding to *p* < 0.05. This result did not lead to the rejection of the null hypothesis, supporting the assumption of normality.

**FIGURE 3:**
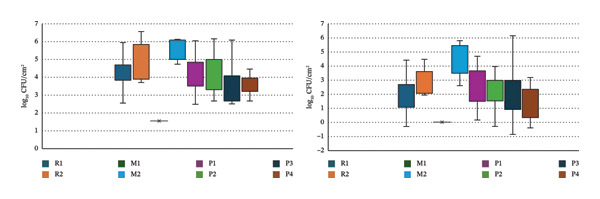
ACC and EntC values distribution among the eight GHEs (R1, R2, M1, M2, P1, P2, P3, and P4).

The one‐way ANOVA on ACC values revealed statistically significant differences across the eight GHEs (Figure 3). Tukey–Kramer’s test further identified the specific GHEs that exhibited significant differences. For a difference to be statistically significant, the calculated q‐score must exceed the corresponding critical value of Tukey’s Q (Table 4). Similarly, the analysis of EntC values in relation to the eight GHEs (Figure 2) also demonstrated significant variations, with Tukey–Kramer’s test highlighting the specific GHEs exhibiting notable differences (Table 4).

**TABLE 4:** *q*‐Scores for ACC and EntC mean values among different GHEs.

ACC *q*‐score	R1	R2	M1	M2	P1	P2	P3	P4
R1		1.72	**5.41**	3.66	0.23	0.35	3.04	2.12
R2	1.72		**6.00**	1.90	2.02	2.10	4.02	3.24
M1	**5.41**	**6.00**		**7.08**	**5.48**	**5.42**	3.97	3.87
M2	3.66	1.90	**7.08**		4.01	4.07	**5.64**	**4.48**
P1	0.23	2.02	**5.48**	4.01		0.16	3.32	2.15
P2	0.35	2.10	**5.42**	4.07	0.16		3.15	2.04
P3	3.04	4.02	3.97	**5.64**	3.32	3.15		0.27
P4	2.12	3.24	3.87	**4.84**	2.15	2.04	0.27	

**EntC q-score**	**R1**	**R2**	**M1**	**M2**	**P1**	**P2**	**P3**	**P4**

R1		2.67	3.32	**5.46**	1.97	0.89	0.01	1.79
R2	2.67		**4.82**	2.39	1.44	2.23	2.69	3.82
M1	3.32	**4.82**		**6.67**	**4.50**	3.92	3.34	1.86
M2	**5.46**	2.39	**6.67**		**4.49**	**5.26**	**5.51**	**6.21**
P1	1.97	1.44	**4.50**	**4.49**		1.30	2.00	3.44
P2	0.89	2.23	3.92	**5.26**	1.30		3.91	2.62
P3	0.01	2.69	3.34	**5.51**	2.00	3.91		1.82
P4	1.79	3.82	1.86	**6.21**	3.44	2.62	1.82	

*Note:* Bold values indicate the statistically significant differences (*p* < 0.05) between specific GHE pairs, where the *q*‐scores exceeded the critical value (4.36).

No statistically significant differences in ACC and EntC values were observed in relation to the sex and age of the wild boars, as well as the hunting method and shooting accuracy. Significant variations were observed for the following variables: the time interval between killing and evisceration, which showed a significant effect only for EntC; the time between evisceration and skinning, which was significant for both ACC and EntC; and the total time from killing to skinning, which was significant for both ACC and EntC (Figure 4).

FIGURE 4(a) ACC and EntC mean values in wild boar samples eviscerated within 3 h of killing and more than 3 h after killing. ACC and EntC were examined independently. (b) ACC and EntC mean values in wild boar samples skinned within 10 h of evisceration and more than 10 h after evisceration. (c) ACC and EntC mean values in wild boar samples skinned within 3.5 h and more than 3.5 h postkilling. A statistically significant difference was observed for (a) EntC (*p* < 0.05); (b, c) ACC and EntC (*p* < 0.05).

**Figure:**
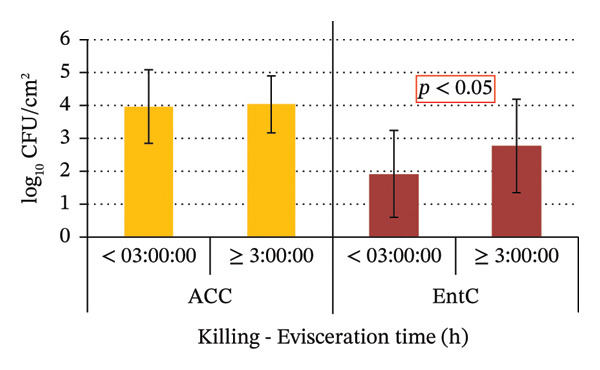
(a)

**Figure:**
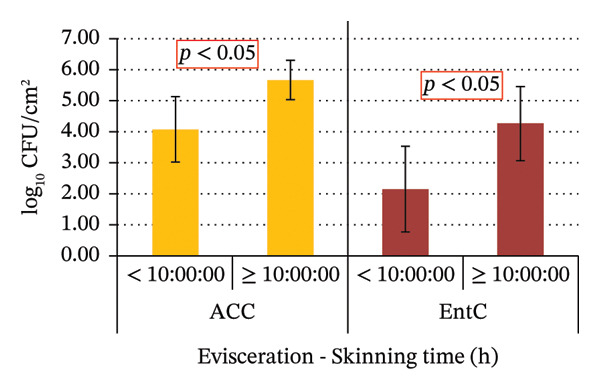
(b)

**Figure:**
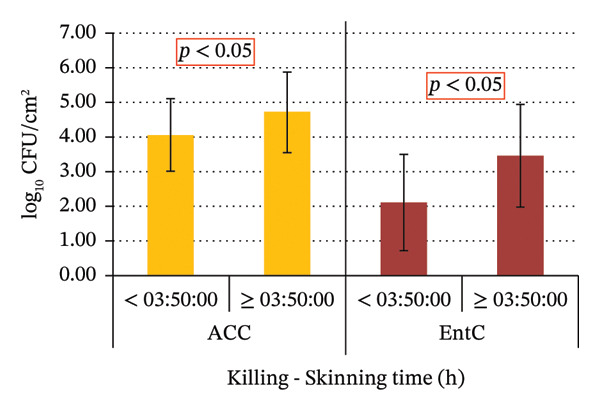
(c)

### 3.3. *Salmonella* Prevalence in Relation to the Recorded Variables

*Salmonella* isolates were detected in 4 out of 115 wild boar carcass sponges, representing a prevalence of 3.48% (95% CI = 0.13–6.83%). Among these isolates, three were identified as *S. enterica* subsp. *enterica,* i.e., two *S.* Coeln and one *S.* Typhimurium, and one was identified as *S. enterica* subsp. *diarizonae* O:50 (z). The variables associated with the *Salmonella* strains are shown in Table 5.

**TABLE 5:** *Salmonella* isolates and their associated recorded variables.

*Salmonella* serovar/subtype	GHE	Sex	Age	Hunting method	Shot accuracy	Killing‐evisceration time	Evisceration‐skinning time	Killing‐skinning time
Coeln	P1	Male	Young	Driven‐hunting	Yes	≥ 3 h	< 10 h	≥ 3.50 h
Coeln	P1	Male	Adult		Yes			< 3.50 h
Typhimurium	P1	Male	Young		Yes			
Diarizonae O:50 (z)	P3	Female	Adult		No data			

**FIGURE 5:**
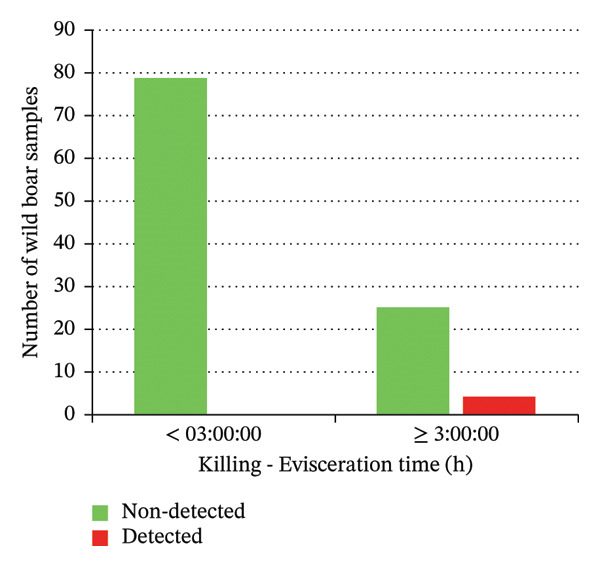
Number of wild boar carcasses in which *Salmonella* was detected or not detected, comparing those eviscerated within 3 h versus those eviscerated after more than 3 h.

A statistically significant difference was observed between positive or negative samples in relation to the time interval between killing and evisceration. Notably, all positive carcasses showed an interval ≥ 3 h (Fisher’s exact test; *p* < 0.05) (Figure 5). No significant differences were observed in any of the other variables analyzed. However, it is noteworthy that three out of the four *Salmonella* isolates were recovered from wild boars skinned in the same GHE.

**FIGURE 6:**
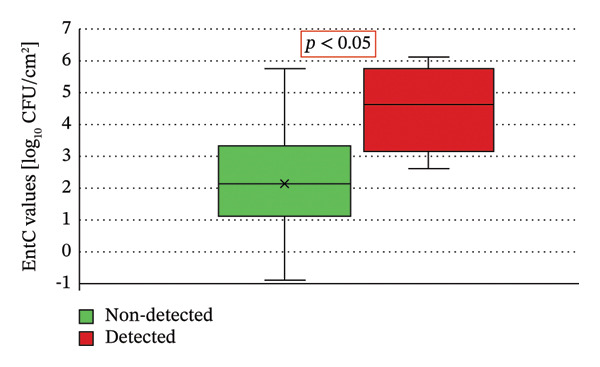
Distribution of EntC values in wild boar carcasses based on Salmonella occurrence. A statistically significant difference was observed (*p* < 0.05).

A statistically significant difference was also observed in the EntC values between wild boar carcasses positive or negative for *Salmonella* spp., as determined by the Mann–Whitney *U* Test (*p* < 0.05) (Figure 6).

### 3.4. AMR in *Salmonella* Isolates

The four *Salmonella* isolates were tested for AMR. *S.* Coeln and *S.* Typhimurium were susceptible to all the antimicrobials tested, while *S. diarizonae* O:50 (z) exhibited resistance to sulfamethoxazole, as determined by EUCAST [34] and CLSI [35] guidelines. The MIC values (μg/mL) are reported in Table 6.

**TABLE 6:** MIC values of the Salmonella strains.

*Salmonella* serovar/subspecies	AMP	CTX	CAZ	MEM	AZI	AMI	GEN	NAL	CIP	COL	TET	TGC	CHL	TMP	SMX
S. Coeln	2	< 0.25	< 0.25	< 0.03	4	< 4	< 0.5	< 4	0.03	< 1	4	< 0.25	< 8	0.5	64
S. Coeln	2	< 0.25	0.5	< 0.03	4	< 4	< 0.5	< 4	0.03	< 1	< 2	< 0.25	< 8	0.5	32
S. Typhimurium	2	< 0.25	0.5	< 0.03	8	< 4	< 0.5	< 4	0.03	< 1	< 2	< 0.25	< 8	0.5	32
S. diarizonae O:50	2	< 0.25	< 0.25	0.06	8	< 4	< 0.5	< 4	0.03	< 1	< 2	0.5	< 8	< 0.25	> 512

### 3.5. Prevalence and Virulence Factors of *E. coli*

*Escherichia coli* strains were isolated from 76 out of 78 wild boar diaphragmatic muscle samples, with a prevalence of 97.43% (95% CI = 93.2–100%).

None of the isolates exhibited phenotypic resistance to third‐generation cephalosporins or carbapenems.

Negative results for all tested virulence genes were observed in 46 out of 76 *E. coli* isolates, corresponding to 60.52% (95% CI = 49.5–71%). The remaining 30 isolates (39.5%, 95% CI = 28.5–50%) tested positive for at least one virulence gene. The *eae* gene, associated with the EPEC pathotype, was detected in only one of 30 isolates (3.33%, 95% CI = 0–9.72%), while 7 of 30 strains (23.3%, 95% CI = 8.3–38.3%) carried the *escV* gene of the LEE PAI, associated with multiple pathotypes. The most frequently detected gene was *astA*, associated with EAEC, found in 26 of 30 *E. coli* isolates (86.7%, 95% CI = 74.7–98.7%). The *aggR* and *pic* genes, both linked to EAEC, were detected in 1 of 30 isolates (3.33%, 95% CI = 0–9.72%) and 3 of 30 isolates (10%, 95% CI = 0–20.7%), respectively. No *stx1* and *stx2* genes were detected; thus, no STEC strains were identified. Similarly, no *E. coli* strains tested positive for the *bfpB*, *elt*, *estIa*, *estIb*, and *invE* genes, excluding the presence of ETEC and EIEC pathotypes. Eight of the 30 *E. coli* isolates (26.6%, 95% CI = 10.8–42.4%) carried more than one virulence gene. Overall, the pathotypes most likely detected in wild boars were atypical EPEC (*eae+* and *bfpB−*) and typical (*aggR+*) and atypical (*aggR−*) EAEC. A summary of the virulence gene patterns is provided in Table 7.

**TABLE 7:** Virulence gene patterns and their respective prevalence in the 30 *E. coli* isolates, along with their probable associated pathotypes and the corresponding GHEs.

No. of *E. coli*	Virulence gene pattern					Prevalence (95% CI)	Probable associated pathotype	GHE
	eae	escV	astA	aggR	plc			
1	+		+			3.33% (0%–9.72%)	aEPEC/aEAEC	P1
1		+				3.33% (0%–9.72%)	Not classifiable	P1
6		+	+			20% (5.7%–34.4%)	aEAEC	R2‐P1‐P2‐P3
1			+	+		3.33% (0%–9.72%)	EAEC	P1
18			+			60% (42.5%–77.5%)	aEAEC	R2‐P1‐P2‐P3‐P4
3					+	10% (0%–20.7%)	aEAEC	P1‐P2

## 4. Discussion

The hygiene conditions during hunting and the subsequent handling of game differ significantly from those kept in slaughterhouses. Thus, the potential for microbial contamination in game meat is much higher than in a controlled slaughterhouse, including the risk of secondary contamination during evisceration, and other manipulations in the field [39]. The ACC and EntC mean values detected in this study were 4.22 and 2.27 Log CFU/cm^2^, respectively. Some Italian studies, evaluating the microbial contamination of wild boar carcasses via sponge sampling, have reported similar ACC and EntC values (4.67 and 2.60 Log CFU/cm^2^[40]; 4.61 and 3.00 Log CFU/cm^2^[41]). Nevertheless, other studies have shown lower (3.21 and 1.32 Log CFU/cm^2^[42]) or higher levels (5.88 and 5.39 Log CFU/cm^2^[43]). Compared to the limits established for farmed pig carcasses by the Regulation (EC) No 2073/2005, with modifications introduced by the Italian State‐Regions Agreement No 41/2016, the ACC and EntC mean values from this study were classified as acceptable. However, due to slaughter and skinning process, it could be more reasonable to evaluate the microbial values of slaughtered wild boars with the process hygiene criteria of skinned animals (i.e., bovines) [42]. When compared to the thresholds for bovine carcasses, the ACC and EntC mean that values of the wild boars tested were also classified as acceptable.

Wild boars are recognized reservoirs of pathogenic microorganisms, including *Salmonella* spp., a multihost pathogen known for its long‐term environmental persistence. The prevalence found in this study was 3.48% (4 out of 115). However, the rates of *Salmonella* in wild boars reported across Italy vary significantly, ranging from negative findings [42, 43] to rates such as 2.5% [44], 3.6% [45], 4.5% [46], 6.27% [47], and up to 35.7% [48]. This variability may be attributed to wild boars distinct microbial populations due to their lack of territorial boundaries and highly variable diets, leading to significant geographical variation [39].

The serovars identified in this study have been frequently isolated from wild boars in Italy [48–51]. *S*. Typhimurium is a frequently detected serovar in wildlife, including wild boars [52], and is also prevalent in farmed species. Its presence is particularly concerning, as it was the second most reported cause of human salmonellosis in the EU in 2023 [20]. Similarly, *S*. Coeln is often identified in wild boars and ranked fifth among serovars responsible for human salmonellosis in 2023, underscoring its potential public health impact [20]. Salmonellosis is an enteric disease and poses notable public health risks, manifesting in enteric fever, gastroenteritis, septicaemia, and focal infections. In Italy, over 3300 cases were documented [20]. *S.* diarizonae O:50 is a serovar commonly isolated from beef, sheep, and snakes [49], but it is also reported in wild boars [52]. While this serovar is rarely implicated in human infections, its prevalence in wildlife warrants attention as a potential zoonotic agent. However, no cases of salmonellosis linked to the consumption of wild boar meat have been reported in Italy [50].

In the present study, *E. coli* was isolated from 97.43% (76/78) of the wild boar diaphragmatic muscle samples. By contrast, another Italian study reported a lower prevalence of 21.8% in fresh wild boar meat [53]. *E. coli* is a Gram‐negative, rod‐shaped bacterium that is ubiquitous in the environment and naturally inhabits the intestines of both animals and humans as a commensal organism. However, some strains of *E. coli* have acquired specific virulence factors, enhancing their ability to adapt to new environments and enabling them to cause a wide range of diseases [23]. Indeed, another significant concern regarding *E. coli* in food‐producing animals, including wild species and products thereof, is the occurrence of pathogenic strains. However, data related to hunting animals and game meat are scarce, despite various *E. coli* pathotypes having been detected in wild boars, with reports from Italy [24, 48] and other European countries [54, 55]. In this study, the *E. coli* isolates were genotypically characterized for the presence of gene markers associated with different pathotypes. Overall, 39.5% (30/76) of the *E. coli* isolates carried at least one virulence gene. In our study, one *E. coli* strain (3.33%) was positive for the *eae* gene, classifying it as atypical EPEC (aEPEC) due to the absence of bundle‐forming pilus gene (*bfp*). The role of aEPEC in human health remains uncertain [56], although some clinical cases, particularly in children, have been documented [57]. Similarly, another Italian study reported a prevalence of 3.4% for aEPEC in wild boar faeces, with the *eae* gene detected in 17.1% of samples [24]. Higher prevalence was observed in wild boar meat, where 32.14% of samples were positive for EPEC [48].

The aEPEC strain identified in this study was also positive for the *astA* gene, classifying it additionally as atypical EAEC (aEAEC). The occurrence of non‐specific profiles, shared by multiple pathotypes, as well as hybrid strains, has become increasingly common and widespread [24, 58]. Notably, only typical EAEC strains carrying the *aggR* gene, which is essential for the expression of other virulence genes, are considered human pathogens [59]. In this study, one typical EAEC (*aggR*+, *astA*+) was identified (3.33%), along with 28 aEAEC strains exhibiting three distinct gene patterns. No STEC, ETEC, and EIEC strains have been detected.

A limitation of this study is related to the negative results concerning STEC detection that may be attributed to the isolation method we used, which primarily targeted commensal *E. coli* isolation followed by strain characterization. EFSA and ECDC [20] recommend the ISO/TS 13136:2012 [36] method for STEC detection, which includes a fundamental initial enrichment screening step.

As established by the Commission Implementing Decision (EU) No 2020/1729 [60], the monitoring of AMR is mandatory for *Salmonella* spp. and indicator *E. coli* in major farmed animal populations and their derived meat products. However, such monitoring is not required for hunted game [61]. In this study, AMR was evaluated in the *Salmonella* and *E. coli* isolates from wild boar carcasses and meat. Similarly to data shown in this study, it is reported in northern Italy low levels of resistance in *Salmonella* serovars, with resistance to sulfamethoxazole detected exclusively in *S. diarizonae*[51]. In contrast, high levels of resistance in *Salmonella* isolates from wild boars have been reported in central Italy, particularly to sulphonamides, trimethoprim, colistin, streptomycin, gentamicin, tetracycline, and third‐generation cephalosporins [49].

All *E. coli* isolates demonstrated phenotypic susceptibility to cephalosporins and carbapenems. In contrast, other Italian studies have identified ESBL‐producing *E. coli* in wild boars, with prevalence rates of 15.96% [62] and 23.3% [63] in faeces, 0.9% in mesenteric lymph nodes [64], and 6.5% in fresh meat [65]. This variability could primarily be attributed to variations in sampling methodologies; however, factors such as dietary differences among wild boars driven by food availability, higher levels of environmental AMR contamination, and proximity to farmed animals and human can also play a role.

All collected data were statistically evaluated comparing counted values with all the variables collected.

The contamination level of wild boars carcasses showed statistically significant difference among the eight GHEs, conversely to data reported by Peruzy et al. [43] suggesting that factors beyond the hunting environment might also play a pivotal role in influencing microbial loads.

No significant difference was reported between values recorded and biometric data such as sex and age of wild boars as highlighted in different Italian studies [42, 43, 46, 47]. Conversely, Ranucci et al., Orsoni et al., and Stella et al. [40, 42, 44] reported significant differences in ACC and EntC levels among age groups, and increasing alongside with animal weight that is a correlated parameter. This suggests that older wild boars may carry higher microbial loads due to the requiring more time and effort to retrieve and transport to the collection point. For what concerning *Salmonella* contamination, some studies have identified a significantly higher prevalence of *Salmonella* in younger animals [66]. Furthermore, Wacheck et al. and Rodas et al. [45, 67] reported higher pathogen prevalence in female wild boars compared to males. These findings may be attributed to behavioural differences as females and juveniles typically form social groups while adult males are more solitary, potentially limiting their exposure to pathogens.

No statistical difference was associated to shot accuracy and to hunting approaches in this study, likely due to over 80% of the wild boars being killed with accurate shot placement. Similarly, other studies have found no significant impact of shooting accuracy on carcass contamination [68]. However, lower microbial loads were observed when a single, effective shot ensured rapid death in wild boars [43].

The time period between killing and evisceration is another critical factor influencing game meat microbiological quality. It has been reported that a 3‐h window is considered critical for minimizing bacterial spread from the gastrointestinal tract [41]. In line with this, the present study found that wild boars eviscerated within 3 h post‐killing exhibited significantly lower carcass EntC microbial contamination, including *Salmonella,* compared to those eviscerated after more than 3 h (*p* < 0.05). The primary source of wild boar carcass contamination is the hide, as the prolonged presence of bristles increases microbial loads on the carcasses [41]. Accordingly, studies recommend prompt skinning to reduce contamination risks [51]. In the present study, the time period between evisceration and skinning significantly influenced ACC and EntC values. In fact, carcasses skinned within 10 h from evisceration showed lower microbial loads compared to those skinned after 10 h (*p* < 0.05). In this study, the longest recorded interval between evisceration and skinning was 60 h, observed in a single sample, which notably exhibited the highest ACC values. The total time between killing and skinning was also considered in this study. The samples of wild boar skinned within 3.5 h from killing showed significantly lower ACC and EntC values compared to those skinned more than 3.5 h after killing. These findings emphasize the potential impact of factors such as geographic location, wild boar populations, hunting practices, and hygiene procedures at different GHEs on carcass contamination levels. These variables likely contribute to the observed differences in microbial prevalence. Monitoring carcass contamination, as well as AMR in hunted game, is crucial, even in the absence of high prevalence, to better understand their environmental spread and impact, through a One Health approach.

## 5. Conclusion

In conclusion, based on the results obtained in this study, it can be stated that despite the numerous variables associated with the hunting process, the overall values for ACC, EntC, and the prevalence of *Salmonella* may be considered “acceptable” under Regulation (EC) No 2073/2005 [13]. However, as previously emphasized, standardized microbiological criteria for hunted game at the European level should be set to monitor the entire hunting process. This should include comprehensive sampling plans, defined microbiological limits, and reference methods. Such measures would facilitate more targeted inspections by the competent authorities and could enhance hunters’ awareness through adequate training to reduce carcass contamination, whether for self‐consumption or local marketing.

Timelines in the hunting process proved to be of critical importance in fact, ensuring the entire process in maximum 4 h guarantees the best conditions to prevent microbial contamination of the carcasses.

However, challenges related to long distances to collection centres or GHEs and impervious geographical areas make adhering to these timelines complex and logistically demanding. Moreover, this study confirmed that wild boars, being free‐ranging animals, can act as reservoirs of pathogens such as *Salmonella* and pathogenic *E. coli*. Although no resistance was detected to critically important antimicrobials (CIAs), resistance to sulfamethoxazole in *Salmonella diarizonae* was identified. These findings underline the importance of more frequent monitoring studies to safeguard both consumer and hunter health, tracking the prevalence of foodborne pathogens and AMR in wildlife.

## Funding

This research did not receive any specific grant from funding agencies in the public, commercial or not‐for‐profit sectors.

Open access publishing facilitated by Universita degli Studi di Parma, as part of the Wiley ‐ CRUI‐CARE agreement.

## Conflicts of Interest

The authors declare no conflicts of interest.

## Data Availability

The data that support the findings of this study are available from the corresponding author upon reasonable request.
